# A Versatile Medium for Cultivating Methanogenic Archaea

**DOI:** 10.1371/journal.pone.0061563

**Published:** 2013-04-17

**Authors:** Saber Khelaifia, Didier Raoult, Michel Drancourt

**Affiliations:** Aix Marseille Université, URMITE, UMR63 CNRS 7278, IRD 198, Inserm 1095, 13005, Marseille, France; University of Groningen, The Netherlands

## Abstract

**Background:**

*Methanobrevibacter smithii, Methanobrevibacter oralis, Methanosphaera stadtmanae, Methanomassilicoccus luminyensis* and *Methanobrevibacter arboriphilicus* have been cultured from human digestive microbiota. Each one of these fastidious methanogenic archaea requires a specific medium for its growth, hampering their routine isolation and the culture.

**Methodology/Principal Findings:**

A new culture medium here referred as SAB medium was optimized and tested to cultivate methanogens associated with human microbiota, as well as two mesophile methanogens *Methanobacterium beijingense* and *Methanosaeta concilii*. It was further tested for the isolation of archaea from 20 human stool specimens including 10 specimens testing positive for PCR detection of *M. smithii*. After inoculating 10^5^ colony-forming-unit archaea/mL or 1 g stool specimen in parallel in SAB medium and reference DSMZ medium in the presence of negative controls, growth of archaea was determined by optical microscopy and the measurement of methane production by gas chromatography. While the negative controls remained sterile, all tested archaea grew significantly more rapidly in SAB medium than in reference medium in 1–3 days (*P*<0.05, Student test). Among PCR-positive stool specimens, 10/10 grew in the SAB medium, 6/10 in DSMZ 119 medium, 5/10 in DSMZ 322 medium and 3/10 in DSMZ 334 c medium. Four out of ten PCR-negative stool specimens grew after a 3-week incubation in the SAB-medium whereas no growth was detected in any of the reference media. 16S rRNA gene sequencing yielded 99–100% sequence similarity with reference *M. smithii* except for one specimen that yielded 99–100% sequence similarity with reference *Methanobrevibacter millerae*.

**Conclusions/Significance:**

SAB medium allows for the versatile isolation and growth of methanogenic archaea associated with human gut microbiota including the archaea missed by inoculation of reference media. Implementation of the SAB medium in veterinary and medical microbiology laboratories will ease the routine culture-based detection of methanogenic archaea in clinical and environmental specimens.

## Introduction

So far, new laboratory techniques for cultivating strict anaerobes, including the technique of Hungate [1], [2] have allowed isolation and further characterization of several new species of the methanogenic archaea associated with human microbiota [3]–[8]. However, isolating methanogenic archaea remains a fastidious process because of the slow growth of these archaea and because of their extreme intolerance to oxygen [9]. Accordingly, an atmosphere consisting of 80% H_2_ and 20% CO_2_ is one of the specific requirements for the optimal growth of these archaea [10]. Mastering the techniques of anaerobic culture is not sufficient to isolate and cultivate methanogens and additional knowledge in nutrient requirements has proved useful to design methanogenic archaea culture media [11].

Some methanogenic archaea, such as *Methanobrevibacter smithii* [7], *Methanobrevibacter oralis* [4] and *Methanobrevibacter arboriphilicus* [12] that we recently isolated from two human stool specimens (S. Khelaifia and M. Drancourt, unpublished data) were reported to require ruminal fluid for growth. *Methanosphaera stadtmanae* [8] has the most restricted energy metabolism of all known human-associated methanogenic archaea, generating methane through the reduction of methanol with H_2_ and being strictly dependent on acetate as a carbon source [13]. Recently, we showed that a selenite/tungstate solution boosted the growth of this archaea [14] and was required for culturing a new methanogen, the *Methanomassilicoccus luminyensis* from human stool specimens [3]. The growth of other archaeal species requires aromatic amino-acid tryptophan, thiamine, pyridoxine, p-aminobenzoic acid (PABA), branched-chain fatty acids, acetate and other unknown factors [15], [16]. Methanogens further require metal ions such as nickel, which is present in F430, hydrogenase and carbon monoxide dehydrogenase [17], [18].

Because of these specific requirements, laboratories are currently preparing one specific culture medium for each one of the various methanogenic archaea species. As a consequence, methanogens are excluded from the routine culture of human gut microbiota, despite the fact that they have been associated with some pathologies including digestive tract diseases, obesity and vaginal infection [19]–[21] and more convincingly periodontitis [22]–[24]. In this study, we aimed to design a new, versatile culture medium that would allow for the culture of all methanogenic archaea.

## Materials and Methods

### Archaea Organisms

*M. smithii* ATCC 35061^T^ DSMZ 861, *M. smithii* DSMZ 2374, *M. smithii* DSMZ 2375, *M. smithii* DSMZ 11975, *M. oralis* DSMZ 7256 ^T^, *M. stadtmanae* ATCC 43021^T^ DSMZ 3091, *M. beijingense* DSMZ 15999 and *M. concilii* DSMZ 2139 purchased from the German Collection of Microorganisms and Cell Cultures (DSMZ, Braunschweig, Germany). The *M. arboriphilicus* strain tested in this study was recently isolated in our laboratory from one human stool specimen. The *M. smithii* strains, *M. arboriphilicus* and *M. beijingense* [6] were grown in liquid medium 119 (http://www.dsmz.de). To cultivate *M. oralis*, medium 119 was modified by the addition of 1 g/L of yeast extract and 1 g/L of peptone, and a 2.5 bar H_2_/CO_2_ (80%-20%) atmosphere was used. Medium 322 (http://www.dsmz.de) was used to cultivate *M. stadtmanae*. Medium 334c was used (http://www.dsmz.de) to cultivate *M. concilii* at 37°C in Hungate tubes (Dutscher, Issy-les-Moulineaux, France) under a 2-bar H_2_/CO_2_ (80%–20%) atmosphere with agitation. *M. luminyensis* CSUR P135^T^ was cultivated using the *Methanobrevibacter* medium (medium 119: http://www.dsmz.de) modified by the addition of methanol and selenite/tungstate solution under 2-bar of H_2_/CO_2_ (80%-20%) atmosphere with agitation [3].

### Clinical Specimens

This study included 20 stool specimens prospectively collected in 20 individuals, in Marseille, France, between July and August 2011. Ten specimens were PCR-positive for *M. smithii,* and ten were PCR-negative. No written consent was needed for this work in accordance with the Law regarding bioethics “n° 2004–800 relative à la bioéthique” published in the “Journal Officiel de la République Française” the 6 August 2004 since no additional sample was taken for the study. This study was approved by the local ethic committee of the Institut Fédératif de Recherche 48, Faculty of Medicine, Marseille, France of 17.02-07 which exempted this study from requiring written informed consent. The samples were analysed anonymously.

Approximately, 1 g of each stool specimen was inoculated in a bottle containing 50 mL of each medium. One decimal dilution was made on the supernatant, in Hungate tubes, to eliminate a maximum of stools and to promote growth only on the culture medium. Vancomycin 100 mg/L and imipenem 100 mg/L (Mylan SAS, Saint Priest, France) and amphotericin B 50 mg/L (Bristol-Myers-Squibb, Rueil-Malmaison, France) were added to the culture medium to eliminate contaminants from intestinal microflora. Growth delay for clinical specimens incubated in the SAB-medium was compared to growth delay in three standard DSMZ culture media 119, 322 and 334c (http://www.dsmz.de).

### SAB Medium

To optimize the SAB-medium, we used a basal medium consisting of components shared by all studied DSMZ media and other media used to isolate and cultivate some methanogenic archaea [3], [5]–[8], [14], [25]. Thereafter, we added to this medium some compounds known to enhanced growth of methanogenic archaea [11], [14], [16]–[18], [26]–[31] and we monitored the effect of each compound on the methanogens’ growth. The definite SAB medium contains the following: NiCl_2_ **.** 6H_2_O, 1.5 mg/L; FeSO_4_ **.** H_2_O, 0.5 mg/L; MgSO_4_ **.** 7H_2_O, 0.8 g/L; KH_2_PO_4_, 0.5 g/L; K_2_HPO_4_, 0.5 g/L; KCl, 0.05 g/L; CaCl_2_ **.** 7H_2_O, 0.05 g/L; NaCl, 1.5 g/L; NH_4_Cl, 1 g/L; MnSO_4_ **.** 7H_2_O, 0.6 mg/L; ZnSO_4_ **.** 7H_2_O, 0.1 mg/L; CuSO_4_ **.** 5H_2_O, 0.02 mg/L; KAl(SO_4_)_2_ **.** 12H_2_O, 0.2 µg/L; H_3_BO_3_, 7 µg/L; CoSO_4_ **.** 7H_2_O, 4 µg/L; Na_2_MoO_4_ **.** 2H_2_O, 0.5 mg/L; Na_2_SeO_3_ **.** 5H_2_O, 3 µg/L; Na_2_WO_4_ × 2H_2_O, 4 µg/L; Nitrilotriacetic acid, 0.15 mg/L; sodium acetate, 1 g/L; trypticase, 2 g/L; yeast extract, 2 g/L; L-cysteine hydrochloride monohydrate, 0.5 g/L; valeric acid, 5 mM; isovaleric acid, 5 mM; 2-methylbutyric acid, 5 mM; isobutyric acid, 6 mM; 2-methyl valeric acid, 5 mM; resazurin, 1 mg/L. The medium was boiled under a nitrogen flux. Bottles were then closed using a lid of aluminum foil and then cooled off to room temperature under N_2_ or 80% N_2_/20%CO_2_ flushing until the medium became transparent. The following compounds were prepared and autoclaved anaerobically under N_2_ and aseptically added to the medium to a final concentration of 2% (v/v): NaHCO_3_, 10%; Na_2_S, 2%; methanol, 4 M; sodium format, 8 M; and vitamin solution [32]. All of the solutions were prepared in anaerobic water, with N_2_/CO_2_ flushing to replace the oxygen [9], [33]. pH was adjusted to 7.5 with 10 M KOH. The culture was incubated using a gas mixture of 80% H_2_+20% CO_2_ at 2.5-bar pressure required for the growth of methanogenic archaea [10], [14]. For all the methanogenic archaea strains, the culture was performed in Hungate tubes incubated at 37°C with agitation.

### Growth Detection

After inoculation of the culture media with 10^5^ colony-forming units (CFU)/mL of archaea or 1 g stool specimen, growth of the methanogens was verified (i) by monitoring the presence of organisms by optical microscopy observations (ii) by measuring methane production using a GC-8A gas chromatograph (Shimadzu, Champs-sur-Marne, France) equipped with a thermal conductivity detector and a Chromosorb WAW 80/100 mesh SP100 column (Alltech, Carquefou, France). N_2_ at a pressure of 100 kPa was used as the carrier gas. The detector and injector temperatures were 200°C and the column temperature was 150°C (iii) by measuring optical density by spectrophotometry (Cary 50 Scan UV-visible spectrophotometer, Agilent Technologies, Paris, France) (iv) by PCR and sequencing of any growing organism (see below). The DSMZ culture medium inoculated with 10^5^ CFU/mL of the corresponding strain was used as positive control culture and non-inoculated culture media were used as negative controls. The growth was monitored and compared for each strain in the corresponding standard DSMZ medium and in the SAB-medium inoculated with the same cell concentration. The experiments were performed in triplicate.

### PCR-sequencing Identification

Identification of detected archaea was performed by PCR amplification and sequencing of the 16S RDNA gene after total DNA extraction as previously described [22]. *Archaea* 16S rDNA was PCR amplified using archaeal primers SDArch0333aS15-5′-TCCAGGCCCTACGGG-3′ and SDArch0958aA19-5′YCCGGCGTTGAMTCCAATT-3′ [23]. Each 50 µl PCR consisted 1× buffer (Qiagen, Courtaboeuf, France), 200 µM each dNTP, 0.2 µM each primer, 2.5 U hotstart *Taq* DNA polymerase (Qiagen) and 5 µl DNA. Archaeal 16S rDNA genes were amplified using the following cycle conditions: 15-min at 95°, 40 cycles of 95°C (30 s), 58°C (45 s) and 72°C (90 s) followed by a 5-min extension step at 72°C. Negative controls consisted in PCR buffer with no DNA. PCR products were purified and sequenced using the BigDye Terminator 1.1 Cycle Sequencing kit and the 3130 genetic analyzer (Applied Biosystems, Villebon-sur-Yvette, France). Sequences were analyzed using the Seqscape program (Applied Biosystems) and similarity values were determined using the online BLAST program at NCBI (www.ncbi.nlm.nih.gov/BLAST/ *)*.

### Statistical Analysis

*P* values were calculated using the non-parametric Kruskal-Wallis test and were used to assess statistical significance for the delay in growth in both culture media. A *P* value <0.05 was indicative of a significant result.

## Results

### Growth Delay Monitoring

**Figure 1:**
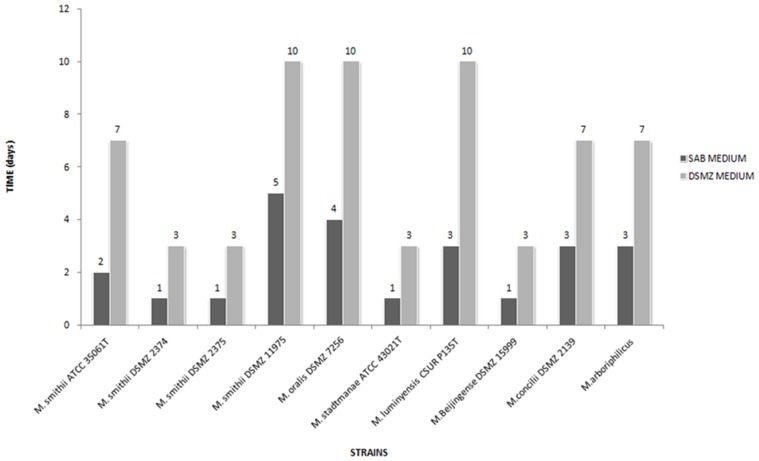
Growth time of ten methanogenic archaea strains growing in culture medium SAB-medium or the standard DSMZ media. (Triplicate experiment).

**Figure 2:**
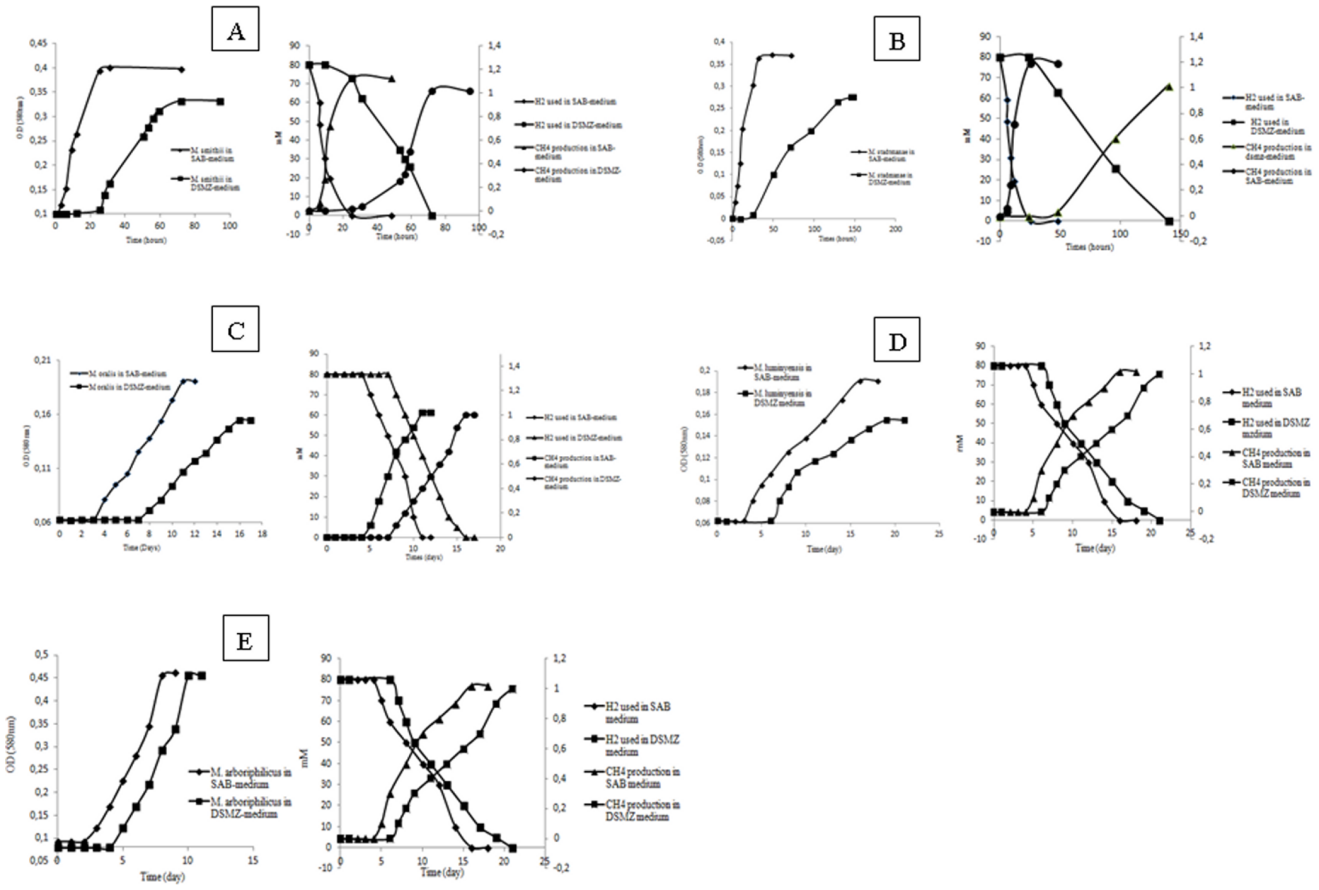
Growth monitoring of the five human-associated archaea strains by monitoring the CH_4_ production and H_2_ used by methanogens growing in culture medium SAB-medium or the standard DSMZ media. A: *Methanobrevibacter smithii;* B: *Methanobrevibacter oralis*; C: *Methanosphaera stadtmanae*; D: *Methanomassilicoccus luminyensis;* E: *Methanobrevibacter arboriphilicus* (Triplicate experiment).

While negative controls remained sterile in all the experiments, all the methanogenic archaea grew as expected, in the recommended standard DSMZ culture medium. All of the methanogenic archaea also grew in the SAB medium. Microscopic observation disclosed organisms with a morphology compatible with the studied archaea organism in each essay and no contaminant. Significant differences were observed in the time required for detecting growth of each methanogenic archaea (Figure 1). *M. smithii* DSMZ 2374, *M. smithii* DSMZ 2375 and *M. smithii* ATCC 35061^T^ grew after 24-hour incubation with a 3-hour doubling time in SAB medium versus 72-hour incubation and a 9-hour doubling time in standard 119 DSMZ medium (*P*<0.05, Student test). *M. smithii* DSMZ 11975 grew after 5-day incubation with a 11-hour doubling time in SAB medium versus 10-day incubation and a 21-hour doubling time in standard 119 DSMZ medium (*P*<0.05). *M. oralis* DSMZ 7256**grew after 3-day incubation with a 18-hour doubling time in SAB medium versus a 7-day incubation and a 21-hour doubling time in standard 119 DSMZ medium modified by the addition of 1 g of yeast extract and 2.5 bar of H_2_/CO_2_ (80%-20%) atmosphere (*P*<0.05). *M. stadtmanae* ATCC 43021^T^ grew after 24-hour incubation with a 3-hour doubling time in SAB medium versus 72-hour and a 9-hour doubling time in standard 322 DSMZ medium (*P*<0.05). *M. luminyensis* CSUR P135^T^ grew after 3-day incubation in SAB medium with a 18-hour doubling time versus 10-day incubation with a 21-hour doubling time in standard 119 DSMZ medium (*P*<0.05). *M. beijingense* DSMZ 15999 grew after 24-hour incubation with a 3-hour doubling time in SAB medium versus 72-hour incubation and a 9–11-hour doubling time in standard 119 DSMZ medium (*P*<0.05). *M. concilii* DSMZ 2139 grew after 3-day incubation with a 9–11-hour doubling time in SAB medium versus 7-day incubation and a 18-hour doubling time in standard 334c DSMZ medium (*P*<0.05). *M. arboriphilicus* grew after 3-day incubation with a 18-hour doubling time in SAB medium versus 5-day incubation and a 18-hour doubling time in standard 119 DSMZ medium (*P*<0.05) (Figure 2). Sequencing the 16S rRNA gene PCR products from all specimens yielded a sequence similarity of 99–100% with the studied archaea strain. Reproducible results were obtained for the triplicate experiments.

### Growth of Clinical Specimens

All PCR-positive stool specimens (10/10) grew in SAB medium after an incubation of one day (six specimens), 3 days (one specimen), 7 days (two specimens) and 10 days (one specimen). After incubation in the 119 DSMZ culture medium, 6/10 specimens yielded detectable growth after incubation of 7 days (five specimens) and 10 days (one specimen). After incubation in the 322 DSMZ culture medium, 5/10 specimens yielded detectable growth after incubation of 7 days (three specimens) and 10 days (two specimens). In 334c DSMZ culture medium, growth was detected for three specimens after an incubation of 7 days (one specimen), 10 days (one specimen) and 15 days (one specimen) (*P*<0.05). As for PCR-negative specimens, 4/10 specimens grew after a 3-week incubation in the SAB-medium whereas they were all sterile in all tested DSMZ culture media (*P*<0.05). In all cases, microscopic observation disclosed organisms with a morphology compatible with *M. smithii* and no contaminant. Sequencing of 16S rRNA gene PCR products from all growing specimens yielded a sequence similarity of 99–100% with the reference *M. smithii* ATCC 35061 except for one PCR-negative specimen which yielded a sequence similarity of 99–100% with the reference *Methanobrevibacter millerae* DSMZ 16643^T^.

## Discussion

The data reported here demonstrate that, under the appropriate temperature and atmosphere conditions, the SAB medium promotes the growth of several methanogenic archaea. This fact has been confirmed since all the negative controls have remained negative in both culture-based and PCR-based experiments; and since all growing organisms have been identified by 16S rRNA gene sequencing. In particular, the medium equally supported the growth of the five methanogens otherwise cultured from human feces specimens in our laboratory. This is a contributive observation as culturing each one of these methanogenic archaea currently requires a species-specific culture medium, thus increasing the time required for the preparation of the culture medium beyond the routine capacity of most laboratories. Moreover, we observed that growth of methanogens was significantly more rapid in the SAB medium than in the reference DSMZ medium.

The design of the SAB medium benefited from careful examination of the media currently used to culture methanogenic archaea and from the knowledge we acquired after the isolation and culture of *M. luminyensis* [3]. In particular, several elements previously reported to be required for growing methanogenic archaea, such as trace metals [27]–[29] were added to SAB-medium at a defined concentration. Indeed, it was reported that the rumen fluid was supplying acetate, branched-chain fatty acids [26] and coenzyme M (2-mercaptoethanesulfonic acid) [34]. The anaerobic conversion of organic matter leads to the intermediate formation of volatile fatty acids, primarily butyrate, propionate and acetate [35]; fecal extract is required for the growth of some methanogens and a volatile fatty acid mixture is highly stimulatory [4]. Some species of the genus *Methanosarcina*, isolated from an anaerobic sludge digester, were originally reported to require an anaerobic sludge supernatant for growth. However, it was found that the sludge supernatant could be replaced with 1 g/L yeast extract, 6 mM bicarbonate-CO_2_ and trace metals [11]. The cysteine used to reduce the medium could also serve as a nitrogen source [11]. *Methanomicrobium mobile* requires the aromatic amino-acid tryptophan and the vitamins thiamine, pyridoxine and PABA [36]. Whereas the composition of yeast extract is variable, the analysis of a typical batch showed that 1 g/L would provide 0.15 µM PABA; accordingly, the vitamin solution we used provided 0.3 µM PABA [32]. These levels of PABA are adequate for the optimal growth of and methanogenesis by archaea of the genera *Methanosarcina* and *Methanomicrobium*. Without the sludge supernatant as a PABA source, it was necessary to increase the concentration of yeast extract and add 1 g/L of peptone to the SAB medium, in addition to a mixture of volatile fatty acids and vitamin solution. This change led to a considerable reduction in the growth time for all of the tested strains (Figure 1).

We propose that the SAB medium provides an opportunity for laboratories to implement the routine culture-based detection of methanogenic archaea for culturing specimens known to contain such methanogens such as periodontal pockets, feces and vaginal discharge [19]–[24]. Using such a medium renders the otherwise fastidious methanogens more amenable to routine practice in microbiology laboratories. Moreover, protocols have been published for the *in vitro* susceptibility testing of these methanogens [37] and the SAB medium could be implemented for such testing.

We developed a unique medium for the rapid growth of methanogens. This medium is now routinely used for the culture-based detection of methanogens from appropriate clinical specimens in our medical microbiology laboratory. Laboratories looking for methanogens of veterinary interest may also want to implement this medium in their routine practices.

### Transparency Declaration

The three co-authors are co-inventors of a patent N/Réf: H52 888 cas 13 FR (MD/SB 12.05.00329) for the SAB medium here reported.
