# Correction: A Versatile Medium for Cultivating Methanogenic Archaea

**DOI:** 10.1371/journal.pone.0312093

**Published:** 2024-10-09

**Authors:** The PLOS ONE Editors

This Correction resolves the issues underlying the Expression of Concern for the linked article [1, 2].

Following the publication of the article and Expression of Concern [1, 2], PLOS investigated concerns pertaining to the reported ethical oversight and the article’s adherence to PLOS research ethics policies.

Specifically, PLOS was concerned that the ethics approval details were not adequately reported in [1], and that the article does not mention Comité de Protection des Personnes (CPP) approval although it involved collection of samples from human participants.

A representative from the Aix-Marseille Université stated that the institutional investigation into the ethics concerns concluded this article meets ethical standards. They stated that this study used stool samples that were collected as part of routine patient care and recovered from a microbiology laboratory in Marseille. They commented that the study received a favourable opinion from the Institut Fédératif de Recherche (IFR) 48 in the document #07–020. They also stated that the stool samples used in this study are considered human waste, and that the study did not require ethics approval from a CPP according to French law.

The IFR 48 #07–020 document was issued on 19 February 2007 for a study titled, “*Détection moléculaire et culture des Archae dans les selles”* and approves the use of anonymised stool samples collected from patients. In light of the documentation provided, the first paragraph of the Clinical Specimens section of the Materials and Methods is updated to:

This study included 20 stool specimens prospectively collected in 20 individuals, in Marseille, France, between July and August 2011. Ten specimens were PCR-positive for *M*. *smithii*, and ten were PCR-negative. No written consent was needed for this work in accordance with the Law regarding bioethics “n° 2004–800 relative à la bioéthique” published in the “Journal Officiel de la République Française” the 6 August 2004 since no additional sample was taken for the study. This study was approved by the local ethics committee of the Institut Fédératif de Recherche 48, Faculty of Medicine, Marseille, France on February 19, 2007 (#07–020), which exempted this study from requiring written informed consent. The samples were analysed anonymously.

With this update, the *PLOS ONE* Editors consider the ethics approval concerns resolved. This Correction supersedes the previous Expression of Concern [2].

Note: The authors did not respond to PLOS’ request for information about inclusion or exclusion criteria that applied in selecting participants for the study. In addition, PLOS identified potential competing interests between the IFR 48 ethics committee that granted the ethics approval and one or more of the article’s authors.
